# Supplementary material for: Correlates of cervical cancer awareness among women aged 30–49 in five sub-Saharan African nations: Evidence from the Demographic and Health Survey (DHS)—2017–2023
Source: PLOS Glob Public Health. 2025 May 7;5(5):e0003344. doi: 10.1371/journal.pgph.0003344 (PMC12057955; doi:10.1371/journal.pgph.0003344)
Supplement: S1 Table — All covariates with p values <0.20 were included in the final multivariate regression model. All formulas were weighted per v005/1000000. *** signifies covariate is significant at p < 0.001; ** signifies covariate is significant at p < 0.01; * signifies covariate is significant at p < 0.05. Confidence intervals and p-values calculated separately for each individual covariate. OR = Odds Ratios. AOR = Adjusted Odds Ratios. NA = Not applicable. (DOCX) [file pgph.0003344.s001.docx]

**S1 Table. Bivariate analyses for cervical cancer awareness for Benin, Cameroon, Madagascar, Mauritania, and Mozambique per Demographic Health Survey 2017-2023, women aged 30-49**

|  | **Benin** | | **Cameroon** | | **Madagascar** | | **Mauritania** | | **Mozambique** | |
| --- | --- | --- | --- | --- | --- | --- | --- | --- | --- | --- |
|  | AOR (95% CI) | AOR (95% CI) | AOR  (95% CI) | *P*-value | AOR (95% CI) | *P*-value | AOR (95% CI) | *P*-value | AOR (95% CI) | *P*-value |
| Age (base: women 30-49) | 1 (0.99-1.03) | P=0.53 | 1.01 (1-1.02) | P=0.2 | 1.01 (0.99-1.02) | P=0.34 | 1 (0.99-1.02) | P=0.62 | 0.95 (0.93-0.98) | P<0.001 |
| Location of housing  Urban  Rural | --  0.38 (0.27-0.54) | P<0.001 | --  0.27 (0.22-0.33) | P<0.001 | ---  0.26 (0.21-0.32) | P<0.001 | ---  0.46 (0.37-0.57) | P<0.001 | ---  0.25 (0.19-0.32) | P<0.001 |
| Current marital status  Never Married  Married Current/Previously | ---  1.17 (1.02-1.33) | P=0.02 | ---  1.07 (1.01-1.13) | P=0.02 | ---  0.96 (0.0-1.01) | P=0.12 | ---  1.16 (1.07-1.25) | P<0.001 | ---  1.09 (1-1.18) | P=0.05 |
| Literacy  Cannot read at all  Able to read | ---  2.24 (1.94-2.6) | P<0.001 | ---  3.27 (2.96-3.6) | P<0.001 | ---  2.65 (2.37-2.95) | P<0.001 | ---  1.7 (1.52-1.91) | P<0.001 | ---  2.89 (2.53-3.31) | P<0.001 |
| Education level  No education  Education (any level) | ---  2.63 (2.24-3.08) | P<0.001 | ---  3.94 (3.52-4.41) | P<0.001 | ---  3.77 (3.32-4.29) | P<0.001 | ---  1.8 (1.58-2.05) | P<0.001 | ---  3.24 (2.74-3.83) | P<0.001 |
| Presence of health insurance  No  Yes | ---  5.65 (3.01-10.59) | P<0.001 | ---  6.08 (3.51-10.55) | P<0.001 | ---  5.8 (4.06-8.28) | P<0.001 | ---  2.4 (1.82-3.17) | P<0.001 | ---  8.53 (3.28-22.17) | P<0.001 |
| Wealth index for Urban/Rural  Lower Class  >Lower Class | ---  1.58 (1.41-1.78) | P<0.001 | ---  1.68 (1.57-1.79) | P<0.001 | ---  1.66 (1.55-1.77) | P<0.001 | ---  1.23 (1.14-1.32) | P<0.001 | ---  1.74 (1.58-1.91) | P<0.001 |
| Frequency of listening to the radio  Not at all  Less than once a week or more | ---  1.63 (1.39-1.91) | P<0.001 | ---  2.25 (2.04-2.47) | P<0.001 | ---  2.26 (2.05-2.49) | P<0.001 | ---  1.24 (1.08-1.43) | P=0.003 | ---  1.43 (1.25-1.62) | P<0.001 |
| Frequency of watching television  Not at all  Less than once a week or more | ---  2.1 (1.79-2.47) | P<0.001 | ---  2.57 (2.34-2.83) | P<0.001 | ---  2.52 (2.24-2.84) | P<0.001 | ---  1.84 (1.61-2.09) | P<0.001 | ---  2.8 (2.43-3.24) | P<0.001 |
| Frequency of reading newspaper or magazine  Not at all  Less than once a week or more | ---  2.56 (2.03-3.22) | P<0.001 | ---  3.01 (2.55-3.56) | P<0.001 | ---  3.16 (2.59-3.86) | P<0.001 | ---  1.77 (1.46-2.15) | P<0.001 | ---  2.98 (2.25-3.93) | P<0.001 |
| Owns a mobile telephone  No  Yes | ---  4.47 (3.2-6.25) | P<0.001 | ---  5.89 (4.92-7.06) | P<0.001 | ---  3.71 (3.13-4.39) | P<0.001 | ---  2.92 (2.25-3.8) | P<0.001 | ---  5.08 (3.88-6.65) | P<0.001 |
| Use of internet  Never  Yes | ---  7.21 (4.65-11.19) | P<0.001 | ---  5.31 (4.39-6.43) | P<0.001 | ---  8.52 (5.95-12.2) | P<0.001 | ---  1.8 (1.47-2.2) | P<0.001 | ---  9.41 (6.76-13.11) | P<0.001 |
| Person who usually decides on respondent’s health care  Respondent alone  Other | ---  0.84 (0.75-0.94) | P=0.002 | ---  0.59 (0.54-0.65) | P<0.001 | ---  0.99 (0.9-1.09) | P=0.82 | ---  0.76 (0.69-0.83) | P<0.001 | ---  0.62 (0.55-0.7) | P<0.001 |
| Distance to the health facility  No problem  Big problem | ---  1.3 (0.9-1.89) | P=0.16 | ---  2.22 (1.88-2.63) | P<0.001 | ---  1.74 (1.44-2.1) | P<0.001 | ---  1.33 (1.09-1.63) | P=0.006 | ---  1.61 (1.23-2.12) | P<0.001 |
| Visited health facility in the last 12 months  No  Yes | ---  1.39 (1.04-1.85) | P=0.024 | ---  2.44 (2.12-2.82) | P<0.001 | ---  1.71 (1.45-2.02) | P<0.001 | ---  1.54 (1.23-1.93) | P<0.001 | ---  3.85 (2.88-5.14) | P<0.001 |
| Tobacco use  Non-smoker  Current smoker | ---  0.52 (0.24-1.17) | P=0.12 | ---  0.9 (0.52-1.55) | P=0.7 | ---  1.32 (0.9-1.93) | P=0.16 | ---  1.28 (0.81-2.01) | P=0.29 | 0.55 (0.3-1) | P=0.05 |
| Hormonal contraceptive use  Not used in past  Yes, used previously | ---  1.45 (1.26-1.66) | P<0.001 | ---  3.49 (2.95-4.14) | P<0.001 | ---  1.47 (1.33-1.61) | P<0.001 | ---  1.45 (1.29-1.64) | P<0.001 | NA | NA |
| Female circumcision/genital cutting: Ever heard of female circumcision?  No  Yes | NA NA | NA NA | NA NA | NA NA | NA NA | NA NA | ---  2.35 (1.45-3.82) | P<0.001 | NA  NA | NA  NA |
| Female circumcision/genital cutting: Ever heard of genital cutting (probed)?  No  Yes | NA  NA | NA  NA | NA  NA | NA  NA | NA  NA | NA  NA | ---  2.38 (0.7-8.14) | P=0.16 | NA  NA | NA  NA |
| Respondent underwent female circumcision/genital mutilation (Base: ever heard of female circumcision) (N= 7495)  No  Yes | NA NA | NA NA | NA NA | NA NA | NA NA | NA NA | ---  0.51 (0.4-0.63) | P<0.001 | NA  NA | NA  NA |
| Emotional abuse: ever experience any emotional violence  No  Yes | NA NA | NA NA | ---  1.3 (1.11-1.74) | P=0.004 | NA NA | NA NA | ---  1.1 (0.8-1.52) | P=0.54 | NA  NA | NA  NA |

**Supplementary Table 1. Bivariate analyses for cervical cancer awareness by individual country for women aged 30-49**. All covariates with p values <0.20 were included in the final multivariate regression model. All formulas were weighted per v005/1000000. *** signifies covariate is significant at p<0.001; ** signifies covariate is significant at p<0.01,; * signifies covariate is significant at p<0.05. Confidence intervals and p-values calculated separately for each individual covariate. OR = Odds Ratios. AOR = Adjusted Odds Ratios.
