# Supplementary material for: Correlates of cervical cancer awareness among women aged 30–49 in five sub-Saharan African nations: Evidence from the Demographic and Health Survey (DHS)—2017–2023
Source: PLOS Glob Public Health. 2025 May 7;5(5):e0003344. doi: 10.1371/journal.pgph.0003344 (PMC12057955; doi:10.1371/journal.pgph.0003344)
Supplement: S2 Table — All covariates with p values <0.20 were included in the final multivariate regression model. All formulas were weighted per v005/1000000. *** signifies covariate is significant at p < 0.001; ** signifies covariate is significant at p < 0.01; * signifies covariate is significant at p < 0.05. Confidence intervals and p-values calculated separately for each individual covariate. OR = Odds Ratios. AOR = Adjusted Odds Ratios. NA = Not applicable. (DOCX) [file pgph.0003344.s002.docx]

**S2 Table. Multivariate weighted analysis for cervical cancer awareness for Benin, Cameroon, Madagascar, Mauritania, and Mozambique per Demographic Health Survey 2017-2023, women ages 15-49.**

|  | **Benin** | | **Cameroon** | | **Madagascar** | | **Mauritania** | | **Mozambique** | |
| --- | --- | --- | --- | --- | --- | --- | --- | --- | --- | --- |
|  | AOR (95% CI) | *P*-value | AOR  (95% CI) | *P*-value | AOR (95% CI) | *P*-value | AOR (95% CI) | *P*-value | AOR (95% CI) | *P*-value |
| Age (base: women 15-49) | 1.03 (1.01-1.04) | P<0.001 | 1.05 (1.04-1.07) | P<0.001 | 1.04 (1.03-1.05) | P<0.001 | 1.03 (1.02-1.04) | P<0.001 | 1.04 (1.02-1.05) | P<0.001 |
| Urban/Rural Residence  Urban  Rural | ---  0.71 (0.52-0.98) | P=0.04 | ---  0.64 (0.5-0.83) | P<0.001 | ---  0.52 (0.41-0.64) | P<0.001 | ---  0.62 (0.46-0.85) | P=0.003 | ---  0.4 (0.3-0.52) | P<0.001 |
| Current marital status  Not married  Married | ---  1.24 (0.9-1.71) | P=0.19 | ---  1.53 (1.24-1.9) | P<0.001 | ---  0.73 (0.57-0.93) | P=0.01 | NA | NA | --  1.93 (1.49-2.49) | P<0.001 |
| Literacy  Cannot read  Can read part. sent.  Can read whole sent.  No card with lang.  Blind/visually impair. | ---  0.65 (0.39-1.1)  0.7 (0.45-1.1)  1.35 (0.42-4.37)  3.1 (0.42-22.9) | P=0.11  P=0.12  P=0.62  P=0.27 | --  1.53 (1.07-2.19)  1.74 (1.24-2.43)  0.86 (0.16-4.77)  1.26 (0.04-38.52) | P=0.02  P=0.001  P=0.86  P=0.89 | ---  1.26 (0.92-1.72)  1.71 (1.29-2.28)  NA  3.61 (0.67-1.95) | P=0.15  P<0.001  NA  P=0.13 | ---  1.56 (1.14-2.14)  1.44(1.07-1.95)  1.12 (2.27-5.54)  5.54 (1.41-21.73) | P=0.006  P=0.02  P=0.89  P=0.01 | ---  1.64 (1.22-2.19)  2.11 (1.59-2.79)  <0.0001 (NA)  <0.0001 (NA) | P=0.0009  P<0.001  P<0.001  P<0.001 |
| Education level  No education  Primary  Secondary  Higher | ---  1.92 (1.37-2.7)  4.26 (2.55-7.11)  5.85 (2.59-13.22) | P<0.001  P<0.001  P<0.001 | ---  2.34 (1.71-3.32)  3.77 (2.49-5.72)  5.25 (2.85-9.67) | P<0.001  P<0.001  P<0.001 | ---  1.75 (1.29-2.39)  2.69 (1.88-3.86)  5.1 (2.79-9.33) | P<0.001  P<0.001  P<0.001 | ---  0.95 (0.74-1.22)  1.4 (1-1.95)  5.82 (2.24-15.14) | P=0.69  P=0.05  P<0.001 | ---  1.17 (0.87-1.57)  1.15 (0.76-1.72)  2.16 (0.82-5.79) | P=0.3  P=0.51  P=0.13 |
| Insurance status  None  Insured | ---  1.68 (0.9-3.14) | P=0.1 | ---  1.56 (0.73-3.31) | P=0.25 | ---  1.45 (0.93-2.24) | P=0.1 | ---  0.95 (0.65-1.39) | P=0.78 | ---  0.83 (0.28-2.51) | P=0.75 |
| Wealth index  Lower Class  Lower Middle Class  Middle Class  Upper Middle Class  Upper Class | ---  1.03 (0.65-1.65)  1.3 (0.83-2.06)  1.5 (0.94-2.42)  1.74 (1.05-2.88) | P=0.89  P=0.25  P=0.09  P=0.03 | ---  0.97 (0.66-1.41)  1.5 (1.02-2.19)  1.69 (1.17-2.42)  1.92 (1.27-2.93) | ---  P=0.86  P=0.04  P=0.005  P=0.002 | ---  1.44 (1.08-1.93)  1.84 (1.36-2.48)  2.1 (1.53-2.9)  2.16 (1.53-3.05) | P=0.01  P<0.001  P<0.001  P<0.001 | ---  1.06 (0.79-1.43)  1.12 (0.8-1.57)  1.24 (0.85-1.82)  1.46 (1.01-2.11) | P=0.68  P=0.51  P=0.26  P=0.04 | ---  1.55 (1.06-2.26)  1.43 (1-2.05)  1.97 (1.37-2.83)  3.47 (2.33-5.17) | P=0.02  P=0.05  P<0.001  P<0.001 |
| Frequency of listening to the radio  Not at all  < once a week  At least once a week | ---  1.45 (1.04-2.01)  1.35 (1.04-1.76) | P=0.03  P=0.03 | ---  1.15 (0.89-1.49)  1.12 (0.88-1.42) | P=0.29  P=0.36 | ---  1.58 (1.31-1.93)  2.41 (1.95-2.94) | P<0.001  P<0.001 | ---  1.26 (0.98-1.63)  0.89 (0.66-1.2) | P=0.07  P=0.45 | ---  1.19 (0.9-1.57)  0.87 (0.7-1.07) | P=0.21  P=0.19 |
| Owns a mobile phone  No  Yes | ---  1.68 (1.25-2.27) | P=0.006 | ---  1.26 (0.99-1.59) | P=0.06 | ---  1.08 (0.91-1.29) | P=0.35 | ---  1.92 (1.44-2.56) | P<0.001 | ---  1.35 (1.08-1.68) | ---  P=0.009 |
| Use of internet  Never  Yes, last 12 mo.  Yes, pre-last 12 mo. | ---  2.17 (1.42-3.31)  1.65 (0.66-4.13) | P<0.001  P=0.28 | ---  1.34 (0.98-1.83)  1.49 (0.9-2.47) | P=0.07  P=0.12 | ---  2.47 (1.82-3.35)  1.85 (1.03-3.31) | P<0.001  P<0.001 | ---  1 (0.75-1.36)  1.43 (0.8-2.54) | P=0.97  P=0.22 | ---  2.17 (1.64-2.87)  3 (1.75-5.14) | P<0.001  P<0.001 |
| Person who usually decides on resp. health care  Resp. alone  Resp. + husb/partner  Husb/partner alone  Some else  Other | ---  1.65 (0.66-4.13)  1 (0.7-1.42)  0.86 (0.62-1.21)  0.81 (0.09-7.69) | P=0.98  P=0.4  P=0.85  P=0.17 | ---  0.91 (0.67-1.23)  0.61 (0.46-0.84)  2.46 (0.51-11.93)  0.4 (0.02-8.59) | P=0.53  P=0.001  P=0.26  P=0.56 | ---  0.95 (0.78-1.14)  1.32 (1-1.73)  2.28 (0.58-10.07)  <0.0001 (NA) | P=0.58  P=0.05  P=0.27  P<0.001 | ---  1.48 (1.07-2.06)  0.9 (0.64-1.26)  0.72 (0.23-2.23)  <0.0001 (NA) | P=0.02  P=0.54  P=0.57  P<0.001 | ---  1.01 (0.79-1.28)  0.57 (0.43-0.77)  0.81 (0.24-2.75)  0.34 (0.03-3.62) | P=0.97  P=0.0001  P=0.73  P=0.37 |
| Distance to the health facility  No problem  Big problem | ---  0.86 (0.63-1.16) | P=0.32 | ---  1.26 (1.06-1.5) | P=0.009 | ---  1.06 (0.87-1.28) | P=0.56 | ---  0.9 (0.71-1.15) | P=0.41 | ---  0.82 (0.65-1.04) | P=0.1 |
| Visited health facility in the last 12 months  No  Yes | ---  1.32 (1.03-1.69) | P=0.03 | ---  1.53 (1.26-1.85) | P<0.001 | ---  1.13 (0.97-1.32) | P=0.11 | ---  1.5 (1.23-1.83) | P<0.001 | ---  2.09 (1.63-2.69) | P<0.001 |
| Female cirum/genital cutting: Ever underwent female circumcision?  No  Yes | NA | NA | NA | NA | NA | NA | ---  0.8 (0.63-1.01) | P=0.09 | NA | NA |
| Emotional abuse: ever experience any emotional violence  No  Yes | NA | NA | ---  1.3 (1.1-1.58) | P=0.009 | NA | NA | --  1.38 (1.06-1.8) | P=0.01 | NA | NA |

**Supplementary Table 2. Multivariate regression analyses for cervical cancer awareness for women aged 15-49**. All covariates with p values <0.20 were included in the final multivariate regression model. All formulas were weighted per v005/1000000. *** signifies covariate is significant at p<0.001; ** signifies covariate is significant at p<0.01,; * signifies covariate is significant at p<0.05. Confidence intervals and p-values calculated separately for each individual covariate. OR = Odds Ratios. AOR = Adjusted Odds Ratios.
