# Supplementary material for: Correlates of cervical cancer awareness among women aged 30–49 in five sub-Saharan African nations: Evidence from the Demographic and Health Survey (DHS)—2017–2023
Source: PLOS Glob Public Health. 2025 May 7;5(5):e0003344. doi: 10.1371/journal.pgph.0003344 (PMC12057955; doi:10.1371/journal.pgph.0003344)
Supplement: Attachment — Submitted filename: Response to Reviewers_PLOS_GH_DJO_05_23_2024_Final.docx [file pgph.0003344.s004.docx]

**Response to Reviewer’s Comments**

**Reviewer #1**:

Thank you for the opportunity to review this paper. The authors have done well to explore the correlates of cervical cancer awareness in SSA. While this is a great paper, there are still some pertinent issues that must be addressed.

Abstract:

1. The conclusion section of the abstract is more or less like a repetition of the results. It will be great to see very practical policy and practice recommendations there.

Thank you for this comment. We revised the results section abstract for repetition and to provide more practical policy recommendations:

*“Given the known association between awareness and screening, targeted efforts to increase awareness among women with lower socioeconomic status has the potential to reduce global cervical cancer disparities. Potential modalities include co-locating cervical cancer screening with public health programs and implementing large-scale telecommunication outreach programs to improve awareness.”*

Additionally, please see response to Reviewer #3, Question #1.

Introduction:

2. In the first paragraph, the authors present the epidemiology of cervical cancer in LMICs. I advise that the authors include some current statistics regarding the incidence, prevalence, and mortalities of cervical cancer in SSA and in the selected countries.

Thank you for this comment. We revised our introduction to provide better descriptive statistics regarding the state of cervical cancer in SSA:

*“Cervical/uterine cancer remains the most common cancer in 48% of SSA nations per World Health Organization’s International Agency for Research on Cancer’s estimates; nearly 117,944 women in SSA were diagnosed in 2022 (age standardized rate (ASR)=33.4, crude rate (CR)=20.5) with approximately 76,140 women passing away (ASR=22.6, CR=13.2).(5-6) Prior studies attribute global disparities in cervical cancer incidence and mortality to differences in human development and gender inequity indices.(7-8) On an individual level, differences in screening rates are a major contributor to this global crisis, as less than 15% of all women ages 30-49 in LMICs have received cervical cancer screening, compared to 84% in high-income countries (HICs)”*

Additionally, we revised our manuscript to specify in-country-level specific incidence rates at the end of the introduction:

*“Herein we analyze the role of various sociodemographic and healthcare variables on cervical cancer awareness among the most recently published DHS surveys from 2017-2023 including Benin, Cameroon, Madagascar, Mauritania, and Mozambique; cervical cancer is the most or second most common cancer in these selected nations, as 701 Beninese women (ASR=18.1), 5,525 Cameroonian women (ASR=33.1), 4,060 Malagasy women (ASR=41.8), 468 Mauritanian women (ASR=28.3), and 5,456 Mozambique women (ASR=47.8) are afflicted with cervical cancer each year”*

In addition, we have added the following references:

5	Sung H, Ferlay J, Siegel RL, et al. Global cancer statistics 2020: GLOBOCAN estimates of incidence and mortality worldwide for 36 cancers in 185 countries. CA Cancer J Clin 2021;71:209–49.

6	Ferlay J, Ervik M, Lam F, Laversanne M, Colombet M, Mery L, Piñeros M, Znaor A, Soerjomataram I, Bray F (2024). Global Cancer Observatory: Cancer Today. Lyon, France: International Agency for Research on Cancer.

7	Ginsburg O, Bray F, Coleman MP, et al. The global burden of women's cancers: a grand challenge in global health. Lancet. 2017 Feb 25;389(10071):847-860. doi: 10.1016/S0140-6736(16)31392-7.

8	Singh GK, Azuine RE, Siahpush M. Global Inequalities in Cervical Cancer Incidence and Mortality are Linked to Deprivation, Low Socioeconomic Status, and Human Development. Int J MCH AIDS. 2012;1(1):17-30. doi: 10.21106/ijma.12. PMID: 27621956; PMCID: PMC4948158.

We also modified paragraph #4 of the discussion section:

*“This finding aligns with previously documented associations between healthcare*

*decision-making and cervical cancer screening uptake among six DHS surveys from*

*2011-2018 in SSA; (44) prior studies also document an inverse relationship between*

*gender inequity and cervical cancer incidence and morality. (8)*

Finally, we modified the last sentence of the first paragraph of the results section:

*“Cervical cancer awareness was limited [34%; 10%-46%] and only 1,274 women across five countries had undergone cervical cancer screening [3%; 0.4%-7%]. Of those tested, 92% were negative/normal, while 4% were positive (n=51) as self-reported per respondents.”*

3. From line 108, the authors introduce the concept of cervical cancer awareness. It will be of importance to let your readers know exactly what you mean by awareness. It is simply having heard of the disease or it goes beyond that?

Thank you for this comment. We agree that definition of awareness is an important point. We revised the following sentence to reflect the definition of awareness utilized in the selected DHS surveys:

*“Cervical cancer awareness -- referring to the understanding or knowledge of cervical cancer in any capacity -- remains one of the most frequent limiting factors to screening across various settings and countries.”*

4. "Categorizing, describing, and elucidating trends in cervical cancer awareness in various SSA ..." Is that the aim of the study? To examine trends? If that is not the case then please revise the sentence.

Thank you for this comment. The primary aim of the study was to describe correlates of cervical cancer awareness and we have amended the following sentence accordingly:

*“Describing correlates for cervical cancer awareness in various SSA nations can inform public health strategies, including the WHO 90-70-90 triple-intervention strategy to reduce the global burden of cervical cancer”*

5. A major concern in this study is the rationale for selecting the three countries. The authors fail to let us appreciate the differences in cervical cancer across the three countries. Moreover, there are more countries with recent DHS data that have cervical cancer as a variable. Why were they not included?

Thank you for this important comment. In this paper, we selected recently published DHS surveys with cervical cancer awareness as a variable to describe covariates of cervical cancer awareness. During this revision, we included Mozambique, a recently published DHS survey that was not available when we began data analysis, and Cameroon.

We have revised our methodology for clarification below:

*“First, all DHS surveys between 2017-2023 were queried for questions on cervical cancer awareness (e.g., search terms “cervical” OR “cervical cancer” OR “cancer”). Ten countries met initial search criteria for further analysis. Each country’s DHS survey was queried via the DHS publicly-accessible Survey Question Database (https://questiondatabase.dhsprogram.com/); additionally, each country’s full survey questionnaire was individually reviewed for questions on cervical cancer awareness via publicly available DHS Summary Reports. As a result, five countries were excluded due to lack of specific questions on cervical cancer awareness including: Burkina Faso (2021); Cote d’Ivorie (2021); Gabon (2019-2021); Ghana (2022); Kenya (2022); Rwanda (2019-2020). Five countries met full inclusion criteria: Benin (2017-2018), Cameroon (2018), Madagascar (2021), Mauritania (2019-20211), and Mozambique (2022-2023). (Figure 1) Women aged 19-45 years old who participated in the DHS from 2017-2022 were included in this analysis, which was modeled off similar studies. (11, 25, 32). In addition, each DHS survey represents one contiguous entity (i.e., the Benin 2017-2018 survey refers to one entry and was n not sub-divided further).”*

We have also inserted Figure 1 per below:

**Figure 1: Survey Inclusion Criteria**

**DHS Surveys from 2017-2023 with questions on cervical cancer screening (10):**

**Inclusion Criteria (5):**

Benin (2017-2018)

Cameroon (2018)

Madagascar (2021)

Mauritania (2019-2021)

Mozambique (2022-2023)

**Excluded Surveys (5):**

Burkina Faso (2021)

Cote d’Ivoire (2021)

Gabon (2019-2021)

Ghana (2022)

Kenya (2022)

Rwanda (2019-2020)

Additionally, we have outlined all DHS surveys with questions on cervical cancer below with rationale for inclusion and exclusion:

| **DHS Survey (Date)** | **Description of questions on cervical cancer:** | **Reason for exclusion/inclusion** |
| --- | --- | --- |
| Benin (2017-2018) | - Have you ever heard of cervical cancer (1423) - Have you heard of tests to detect cervical cancer (1424) - Has a doctor or other health care professional ever tested you for cervical cancer (1426) - When was your last cervical cancer test (1427) - What was the result of your last cervical cancer test (1428) | - Included |
| Cameroon (2018) | - Have you heard of cervical cancer? (1223) - Have you heard of any test for cervical cancer? (1224) - Has a doctor of other healthcare worker ever tested you for cervical cancer? (1226) - How many years ago was your last test for cervical cancer? (1227) - What was the result of your last test for cervical cancer? (1228) | - Included |
| Burkina Faso (2021) | - Has a doctor or other healthcare worker ever tested you for cervical cancer? (1105) | - Not included: no questions on cervical cancer awareness |
| Cote d’Ivoire (2021) | - Has a doctor or other healthcare worker ever tested you for cervical cancer? (1105) | - Not included: no questions on cervical cancer awareness |
| Gabon (2019-2021) | - Has a doctor or other healthcare worker ever tested you for cervical cancer? (1105) | - Not included: no questions on cervical cancer awareness |
| Ghana (2022) | - Has a doctor or other healthcare worker ever tested you for cervical cancer? (1105) | - Not included: no questions on cervical cancer awareness |
| Kenya (2022) | - Has a doctor or other healthcare worker ever tested you for cervical cancer? (1105) - Have you ever been told by a doctor or other healthcare worker that you have cervical cancer? (1105A) - Have you received any treatment for cervical cancer? (1105B) | - Not included: no questions on cervical cancer awareness |
| Madagascar (2021) | - Have you ever heard of a test for cervical cancer? (1323) - Have you heard of any test for cervical cancer? (1324) - Has a doctor or other healthcare worker ever tested you for cervical cancer? (1326) - When was your last test for cervical cancer? (1327) - What was the result of your last test for cervical cancer? (1228) - Have you ever been told by a doctor or other healthcare worker that you have cervical cancer? (1105A) - Have you received any treatment for cervical cancer? (1105B) | - Included |
| Mauritania (2019-2021) | - Have you ever heard of a test for cervical cancer? (1323) - Have you heard of any test for cervical cancer? (1324) - Has a doctor or other healthcare worker ever tested you for cervical cancer? (1326) - When was your last test for cervical cancer? (1327) - What was the result of your last test for cervical cancer? (1228) - Have you ever been told by a doctor or other healthcare worker that you have cervical cancer? (1105A) - Have you received any treatment for cervical cancer? (1105B) | - Included |
| Mozambique (2022-2023) | - Have you ever heard of a test for cervical cancer? (1103B) - Has a doctor of other healthcare worker ever tested you for cervical cancer? (1226) - How many years ago was your last test for cervical cancer? (1227) - What was the result of your last test for cervical cancer? (1228) - Have you received any treatment for cervical cancer? | - Included |
| Rwanda (2019-2020) | - Do you have a card where HPV vaccinations against cervical cancer are written down? | - Not included: no questions on cervical cancer awareness |

Methods:

6. The authors must include more explanatory variables. Variables such as risky sexual behaviors, having some chronic diseases, having visited the healthcare facility, autonomy in healthcare decision-making are available in the DHS and have been found to influence awareness levels. As such, the authors must run the analysis again taking into consideration all of these possible correlates.

Thank you for this comment. We expanded our multivariate analyses to include two additional important variables that influence cervical cancer awareness: V394 (“Have you visited a health facility during the last 12 months?”) and V467D (“Distance to the healthcare facility”). We have revised our bivariate and multivariate results correspondingly (Tables 1-3). In addition, our analyses include additional variables on autonomy in healthcare decision-making (“person who usually decides on respondent’s healthcare”) as well as other healthcare behaviors (Tables 1-3).

We also acknowledge that several additional medical and sociodemographic variables influence cervical cancer rates in certain settings. In this regard, we previously ran both bivariate and multivariate analyses with various chronic diseases and health factors including but not limited to: tobacco use (i.e., does not smoke, some days, every day), body mass index (sub-divided into >/= 25 kg/m^2^), age at first birth (>19 or </= 19 years old), number of children (>2 or </=2), and hormonal contraceptive use (no, yes outside of calendar year, yes in calendar year), the breakdown of which was described under “health behaviors” in Table 1. After running several models and further analysis, we decided to remove these variables given significant collinearity in multivariate analyses, p-values >0.20, and lack of influence on cervical cancer awareness, among other reasons. Decisions on variable selection were made based on the following references:

30	Bursac Z, Gauss CH, Williams DK, Hosmer DW. Purposeful selection of variables in logistic regression. Source Code Biol Med. 2008;3:17.

31	Shrestha N. Detecting multicollinearity in regression analysis. American Journal of Applied Mathematics and Statistics. 2020 Jun 16;8(2):39-42.

Finally, we also considered adding HIV variables to the dataset in this revision. However,

the identification of HIV positive patients was not available within the requested datasets

and requires additional approvals for patient privacy. Additionally, while HIV is an

important risk factor for cervical cancer, we felt that it is not a predictor of a cervical

cancer awareness. Ultimately, we felt that HIV positive individuals’ awareness of

cervical cancer was beyond the scope of our current project but would be a valuable

secondary or follow up project.

Last, please see the response to Reviewer #3, Question #1.

7. Why do you have separated analysis for each country? Were the data not appended? The study aims to examine the correlates in SSA. This means that the data must be appended and the country and survey years accounted for in the model.

In-country analyses are separated in Tables 2A-2E in bivariate analysis and Table 3 in multivariate analysis, and we have revised all of these results in the text.

Pooled multi-survey meta-analyses were not completed in this project given significant clinical and environmental heterogeneity between Benin, Cameroon, Mauritania, Madagascar, and Mozambique, which would make it challenging to make any meaningful conclusions from pooled data. Additionally, the cross-sectional nature of the DHS surveys limits any causal inference.

This decision was made based on expert opinion and the following review article:

Lensen S. “When to pool data in a meta-analysis (and when not to)?” Fertil Steril. 2023 Jun;119(6):902-903. doi: 10.1016/j.fertnstert.2023.03.015. Epub 2023 Mar 21. PMID: 36948444.

8. The tables presented show the bivariable/bivariate. Where is the multivariable/multivariate results?

Thank you for this comment. We conducted multivariate analyses in Table 3 (located on page 13 within the text). Covariates with p values <0.20 in bivariate analysis met inclusion criteria for inclusion in our logistic multivariable regression model and our model was refined after further tests for collinearity.

Please see Response to Reviewer #3, Question #3.

Please see below for revised multivariate analysis:

**TABLE 3. Multivariate weighted analysis for cervical cancer awareness for Benin, Cameroon, Madagascar, Mauritania, and Mozambique per Demographic Health Survey 2017-2023.**

|  | **Benin** | | **Cameroon** | | **Madagascar** | | **Mauritania** | | **Mozambique** | |
| --- | --- | --- | --- | --- | --- | --- | --- | --- | --- | --- |
|  | AOR (95% CI) | *P*-value | AOR  (95% CI) | *P*-value | AOR (95% CI) | *P*-value | AOR (95% CI) | *P*-value | AOR (95% CI) | *P*-value |
| Age (base: women 15-49) | 1.03 (1.01-1.04) | P<0.001 | 1.05 (1.04-1.07) | P<0.001 | 1.04 (1.03-1.05) | P<0.001 | 1.03 (1.02-1.04) | P<0.001 | 1.04 (1.02-1.05) | P<0.001 |
| Urban/Rural Residence  Urban  Rural | ---  0.71 (0.52-0.98) | P=0.04 | ---  0.64 (0.5-0.83) | P<0.001 | ---  0.52 (0.41-0.64) | P<0.001 | ---  0.62 (0.46-0.85) | P=0.003 | ---  0.4 (0.3-0.52) | P<0.001 |
| Current marital status  Not married  Married | ---  1.24 (0.9-1.71) | P=0.19 | ---  1.53 (1.24-1.9) | P<0.001 | ---  0.73 (0.57-0.93) | P=0.01 | NA | NA | --  1.93 (1.49-2.49) | P<0.001 |
| Literacy  Cannot read  Can read part. sent.  Can read whole sent.  No card with lang.  Blind/visually impair. | ---  0.65 (0.39-1.1)  0.7 (0.45-1.1)  1.35 (0.42-4.37)  3.1 (0.42-22.9) | P=0.11  P=0.12  P=0.62  P=0.27 | --  1.53 (1.07-2.19)  1.74 (1.24-2.43)  0.86 (0.16-4.77)  1.26 (0.04-38.52) | P=0.02  P=0.001  P=0.86  P=0.89 | ---  1.26 (0.92-1.72)  1.71 (1.29-2.28)  NA  3.61 (0.67-1.95) | P=0.15  P<0.001  NA  P=0.13 | ---  1.56 (1.14-2.14)  1.44 (1.07-1.95)  1.12 (2.27-5.54)  5.54 (1.41-21.73) | P=0.006  P=0.02  P=0.89  P=0.01 | ---  1.64 (1.22-2.19)  2.11 (1.59-2.79)  <0.0001 (NA)  <0.0001 (NA) | P=0.0009  P<0.001  P<0.001  P<0.001 |
| Education level  No education  Primary  Secondary  Higher | ---  1.92 (1.37-2.7)  4.26 (2.55-7.11)  5.85 (2.59-13.22) | P<0.001  P<0.001  P<0.001 | ---  2.34 (1.71-3.32)  3.77 (2.49-5.72)  5.25 (2.85-9.67) | P<0.001  P<0.001  P<0.001 | ---  1.75 (1.29-2.39)  2.69 (1.88-3.86)  5.1 (2.79-9.33) | P<0.001  P<0.001  P<0.001 | ---  0.95 (0.74-1.22)  1.4 (1-1.95)  5.82 (2.24-15.14) | P=0.69  P=0.05  P<0.001 | ---  1.17 (0.87-1.57)  1.15 (0.76-1.72)  2.16 (0.82-5.79) | P=0.3  P=0.51  P=0.13 |
| Insurance status  None  Insured | ---  1.68 (0.9-3.14) | P=0.1 | ---  1.56 (0.73-3.31) | P=0.25 | ---  1.45 (0.93-2.24) | P=0.1 | ---  0.95 (0.65-1.39) | P=0.78 | ---  0.83 (0.28-2.51) | P=0.75 |
| Wealth index  Lower Class  Lower Middle Class  Middle Class  Upper Middle Class  Upper Class | ---  1.03 (0.65-1.65)  1.3 (0.83-2.06)  1.5 (0.94-2.42)  1.74 (1.05-2.88) | P=0.89  P=0.25  P=0.09  P=0.03 | ---  0.97 (0.66-1.41)  1.5 (1.02-2.19)  1.69 (1.17-2.42)  1.92 (1.27-2.93) | ---  P=0.86  P=0.04  P=0.005  P=0.002 | ---  1.44 (1.08-1.93)  1.84 (1.36-2.48)  2.1 (1.53-2.9)  2.16 (1.53-3.05) | P=0.01  P<0.001  P<0.001  P<0.001 | ---  1.06 (0.79-1.43)  1.12 (0.8-1.57)  1.24 (0.85-1.82)  1.46 (1.01-2.11) | P=0.68  P=0.51  P=0.26  P=0.04 | ---  1.55 (1.06-2.26)  1.43 (1-2.05)  1.97 (1.37-2.83)  3.47 (2.33-5.17) | P=0.02  P=0.05  P<0.001  P<0.001 |
| Frequency of listening to the radio  Not at all  < once a week  At least once a week | ---  1.45 (1.04-2.01)  1.35 (1.04-1.76) | P=0.03  P=0.03 | ---  1.15 (0.89-1.49)  1.12 (0.88-1.42) | P=0.29  P=0.36 | ---  1.58 (1.31-1.93)  2.41 (1.95-2.94) | P<0.001  P<0.001 | ---  1.26 (0.98-1.63)  0.89 (0.66-1.2) | P=0.07  P=0.45 | ---  1.19 (0.9-1.57)  0.87 (0.7-1.07) | P=0.21  P=0.19 |
| Owns a mobile phone  No  Yes | ---  1.68 (1.25-2.27) | P=0.006 | ---  1.26 (0.99-1.59) | P=0.06 | ---  1.08 (0.91-1.29) | P=0.35 | ---  1.92 (1.44-2.56) | P<0.001 | ---  1.35 (1.08-1.68) | ---  P=0.009 |
| Use of internet  Never  Yes, last 12 mo.  Yes, pre-last 12 mo. | ---  2.17 (1.42-3.31)  1.65 (0.66-4.13) | P<0.001  P=0.28 | ---  1.34 (0.98-1.83)  1.49 (0.9-2.47) | P=0.07  P=0.12 | ---  2.47 (1.82-3.35)  1.85 (1.03-3.31) | P<0.001  P<0.001 | ---  1 (0.75-1.36)  1.43 (0.8-2.54) | P=0.97  P=0.22 | ---  2.17 (1.64-2.87)  3 (1.75-5.14) | P<0.001  P<0.001 |
| Person who usually decides on resp. health care  Resp. alone  Resp. + husb/partner  Husb/partner alone  Some else  Other | ---  1.65 (0.66-4.13)  1 (0.7-1.42)  0.86 (0.62-1.21)  0.81 (0.09-7.69) | P=0.98  P=0.4  P=0.85  P=0.17 | ---  0.91 (0.67-1.23)  0.61 (0.46-0.84)  2.46 (0.51-11.93)  0.4 (0.02-8.59) | P=0.53  P=0.001  P=0.26  P=0.56 | ---  0.95 (0.78-1.14)  1.32 (1-1.73)  2.28 (0.58-10.07)  <0.0001 (NA) | P=0.58  P=0.05  P=0.27  P<0.001 | ---  1.48 (1.07-2.06)  0.9 (0.64-1.26)  0.72 (0.23-2.23)  <0.0001 (NA) | P=0.02  P=0.54  P=0.57  P<0.001 | ---  1.01 (0.79-1.28)  0.57 (0.43-0.77)  0.81 (0.24-2.75)  0.34 (0.03-3.62) | P=0.97  P=0.0001  P=0.73  P=0.37 |
| Distance to the health facility  No problem  Big problem | ---  0.86 (0.63-1.16) | P=0.32 | ---  1.26 (1.06-1.5) | P=0.009 | ---  1.06 (0.87-1.28) | P=0.56 | ---  0.9 (0.71-1.15) | P=0.41 | ---  0.82 (0.65-1.04) | P=0.1 |
| Visited health facility in the last 12 months  No  Yes | ---  1.32 (1.03-1.69) | P=0.03 | ---  1.53 (1.26-1.85) | P<0.001 | ---  1.13 (0.97-1.32) | P=0.11 | ---  1.5 (1.23-1.83) | P<0.001 | ---  2.09 (1.63-2.69) | P<0.001 |
| Female cirum/genital cutting: Ever underwent female circumcision?  No  Yes | NA | NA | NA | NA | NA | NA | ---  0.8 (0.63-1.01) | P=0.09 | NA | NA |
| Emotional abuse: ever experience any emotional violence  No  Yes | NA | NA | ---  1.3 (1.1-1.58) | P=0.009 | NA | NA | --  1.38 (1.06-1.8) | P=0.01 | NA | NA |

**Table 3. Multivariate regression analyses for cervical cancer awareness**. All covariates with p values <0.20 were included in the final multivariate regression model. All formulas were weighted per v005/1000000. *** signifies covariate is significant at p<0.001; ** signifies covariate is significant at p<0.01,; * signifies covariate is significant at p<0.05. Confidence intervals and p-values calculated separately for each individual covariate. OR = Odds Ratios. AOR = Adjusted Odds Ratios.

Results:

9. I advise that the authors append the data and then use a multilevel logistic regression analysis.

Thank you for this comment. Please see answer to #8 above for discussion regarding multivariate regression analysis. In addition, we have revised Tables 2A-E and Table 3 in the text.

10. With the critical analytical issues raised, I cannot review the discussion now. As running the analysis again is likely to change the results.

No response indicated.

**Reviewer #3:**

Introduction:

1. The introduction needs to provide more comprehensive information about cervical cancer in Africa. Notably, 18 out of the 20 countries with the highest burden are in Africa (WHO). This underscores the significance of addressing the disease in this region. In addition of the following challenges in Africa:

• Vaccination: Weak vaccination programs lead to low coverage and effectiveness.

• Interplay with HIV: High HIV prevalence in Africa exacerbates cervical cancer risks.

• Awareness: General awareness of cervical cancer is very poor.

• Screening: Limited access to screening leads to late diagnosis.

• Access to Care: Difficulties in accessing care due to cost and logistical issues.

Thank you for this insightful comment. We revised our introduction to reflect various challenges more comprehensively towards cervical cancer screening and care delivery in sub-Saharan Africa:

*“Notably, global efforts to reduce the burden cervical cancer should address screening, treatment, access to care, vaccination uptake programs, care follow-up, and the interplay of HIV and cervical cancer; however, such analysis is beyond the scope of this project. Herein we analyze the role of various sociodemographic and healthcare variables on cervical cancer awareness in among the most recently published DHS surveys from 2017-2023 including Benin, Cameroon, Madagascar, Mauritania, and Mozambique; cervical cancer is the most or second most common cancer in these selected nations, as 701 Beninese women (ASR=18.1), 5,525 Cameroonian women (ASR=33.1), 4,060 Malagasy women (ASR=41.8), 468 Mauritanian women (ASR=28.3), and 5,456 Mozambique women (ASR=47.8) are afflicted with cervical cancer each year. (5-6)”*

In addition, we added the following sentences to our discussion section, paragraph #6:

*“Importantly, addressing cervical cancer disparities in SSA requires a multi-faceted approach addressing disease awareness, primary prevention (i.e., HPV-16 and HPV-18 vaccinations), and secondary prevention (i.e., screening and treatment). While beyond the scope of this paper, we believe efforts targeted to increase awareness should be partnered with primary and secondary prevention efforts for maximal efficacy”*

*“Last, efforts at increasing awareness and screening should partner with broader capacity-building efforts.(63) Potential modalities include “sandwich fellowships”, post-graduate training programs in HICs bookended with training in home LMIC institution.(64) Special consideration should focus on capacity building in each oncologic-subspeciality including gynecologic oncology, medical oncology, radiation oncology, and pathology; indeed, one prior study estimates there are only eleven pathologists, three medical oncologists and three gynecologic oncologists among the 33 million population of Mozambique.(63) Anecdotally, we report a similar post-graduate capacity-building collaboration between Fred Hutch Cancer Center and the Uganda Cancer Center resulting in training of six Ugandan medical oncologists. Such capacity-building efforts play an essential role in reducing the global burden of cervical cancer..”*

We added the following references in support:

63	Batman S, Rangeiro R, Monteiro E, Changule D, Daud S, Ribeiro M, Tsambe E, Bila C, Osman N, Carrilho C, Neves A, Atif H, De Jesus C, Mariano A, Moretti-Marques R, Vieira M, Fontes-Cintra G, Lopes A, Batware JC, Luis E, Grover S, Baker E, Fellman B, Montealegre J, Castle PE, Jeronimo J, Chiao E, Lorenzoni C, Schmeler K, Salcedo MP. Expanding Cervical Cancer Screening in Mozambique: Challenges Associated With Diagnosing and Treating Cervical Cancer. JCO Glob Oncol. 2023 Sep;9:e2300139. doi: 10.1200/GO.23.00139. PMID: 37824802; PMCID: PMC10664858.

64	Kassam F, Damji KF, Kiage D, Carruthers C, Kollmann KH. The Sandwich fellowship: a subspecialty training model for the developing world. Acad Med. 2009 Aug;84(8):1152-60. doi: 10.1097/ACM.0b013e3181acf95c. PMID: 19638787.

Methodology:

2. Clarify if data from two different surveys were combined in Benin and, if so, explain the rationale behind it.

Thank you for this comment. One survey was used from Benin, the 2017-2018 Demographics Health Survey (DHS). The survey spanned both 2017 and 2018 but represents one contiguous survey entity per the DHS team; data is unable to be broken down further by year.

We added the following sentence for clarification to our methodology:

*“In addition, each DHS survey represents one contiguous entity (i.e., the Benin 2017-2018 survey refers to one entry and is unable to be sub-divided further).”*

In addition, after further review, the Mauritania DHS survey was conducted between 2019-2021, which we clarified in the text.

3. Address concerns regarding collinearity, as the data may represent the same population.

Thank you for this comment. We agree that collinearity is crucial to address and have detailed our approach towards collinearity below:

*“Purposeful selection of all covariates (i.e., covariates with a p-value <0.2 in bivariate analysis) were included in the final regression models.(34) All models were tested for collinearity with variance inflation factor (VIF) >5 as a barometer for significant collinearity.(35) Statistical code was adapted from several open-source R platforms and informative DHS guides produced by USAID.(31, 33) In our multivariate models, only one FGC/M and IPV variable were ultimately included given collinearity”*

We utilized the following references in support of our methodology in regards to purposeful selection and collinearity:

34	Bursac Z, Gauss CH, Williams DK, Hosmer DW. Purposeful selection of variables in logistic regression. Source Code Biol Med. 2008;3:17.

35	Shrestha N. Detecting multicollinearity in regression analysis. American Journal of Applied Mathematics and Statistics. 2020 Jun 16;8(2):39-42.

Findings:

4. The presentation of health insurance data in Benin is unclear. Simplify the table for better clarity, ensuring percentages add up correctly.

Thank you for this comment. We have clarified the percentages in the text per below and revised Tables 2B-E in the text:

**TABLE 2A. Bivariate analysis for association between SES and demographic characteristics and cervical cancer awareness per Benin Demographic Health Survey, 2017-2018.**

| **Characteristic** | **Sample (n)** | **No. aware of cervical cancer** | ***Odds ratios (OR);***  ***P-values*** |
| --- | --- | --- | --- |
| **Sociodemographic factors:** |  |  |  |
| Age (base: women 15-49)  15-19  20-24  25-29  30-34  35-39  40-44  45-49 | 1623 (21%)  1422 (18%)  1488 (19%)  1029 (13%)  942 (12%)  613 (8%)  595 (8%) | 102  159  179  107  99  74  63 | (Ref)  1.86 (1.4-2.47)***  2.06 (1.54-2.76)***  1.67 (1.24-2.24)***  1.75 (1.28-2.39)***  1.96 (1.4-2.71)***  1.74 (1.19-2.54)** |
| Location  Urban  Rural | 3,431 (445%)  4,281 (56%) | 519  264 | (Ref)  0.39 (0.33-0.459)*** |
| Current marital status  Never in union  Married  Living with partner  Widowed  Divorced  No longer living together/separated | 1918 (25%)  4255 (55%)  1151 (15%)  149 (2%)  60 (1%)  179 (2%) | 221  373  128  14  13  34 | (Ref)  0.77 (0.64-0.93)**  0.98 (0.77-1.24)  0.72 (0.37-1.27)  1.65 (0.79-3.14)  1.9 (1.25-2.82)** |
| Literacy  Cannot read at all  Able to read only parts of sent.  Able to read whole sentence | 5,028 (65%)  561 (7%)  2,085 (27%) | 286  47  446 | (Ref)  1.41 (0.998-1.96)*  4.2 (3.57-4.94)*** |
| Education level  No education  Primary  Secondary  Higher | 4236 (55%)  1516 (20%)  1773 (23%)  187 (2%) | 213  137  336  97 | (Ref)  1.84 (1.47-2.31)***  4.24 (3.52-5.12)***  22.48 (16.09-31.49)*** |
| Presence of health insurance  No  Yes | 7628 (9%)  84 (1%) | 747  36 | (Ref)  7.01 (4.35-11.15)*** |
| Wealth index  Lower Class  Lower Middle Class  Middle Class  Upper Middle Class  Upper Class | 1468 (19%)  1528 (20%)  1359 (18%)  1538 (20%)  1819 (24%) | 55  70  99  146  413 | (Ref)  1.22 (0.85-1.78)  2.07 (1.48-2.95)***  2.8 (2.04-3.92)***  7.11 (5.31-9.74)*** |
| Frequency of listening to the radio  Not at all  Less than once a week  At least once a week | 3311 (43%)  1648 (21%)  2753 (36%) | 195  169  419 | (Ref)  1.85 (1.49-2.31)***  2.79 (2.33-3.36)*** |
| Owns a mobile telephone (Ref: no)  Yes | 3936 (51%) | 620 | 0.99 (0.98-1.01) |
| Use of internet  Never  Yes, last 12 months  Yes, before last 12 months | 7117 (92%)  539 (7%)  56 (1%) | 546  228  9 | (Ref)  9.12 (7.46-11.14)***  2.43 (1.11-4.75)* |
| Person who usually decides on respondent’s health care  Respondent alone  Respondent + husband/partner  Husband/partner alone | 613 (11%)  1873 (35%)  2890 (54%) | 72  219  206 | (Ref)  1.08 (0.81-1.46)  0.61 (0.46-0.83)** |

**Table 2A, Bivariate analysis for association between SES and demographic characteristics and cancer screening per Benin Demographic Health Survey, 2017-2018.** *** signifies covariate is significant at p<0.001; ** signifies covariate is significant at p<0.01,; * signifies covariate is significant at p<0.05. Confidence intervals and p-values calculated separately for each individual covariate. SES = Socioeconomic Status. OR = Odds Ratios.

5. Table 2A should focus on identifying factors associated with unawareness of cervical cancer, as this is the central issue being addressed.

Thank you for this comment.

Our variables selected in Table 2A include sociodemographic variables and two additional social health-behavior variables: intimate partner violence and female genital mutilation/circumcision. Each selected variable was associated with awareness (or un-awareness) depending on the polarity.

Sociodemographic variables (age, location, marital status, literacy, education, presence of health insurance, wealth index, radio/telephone/internet usage, healthcare decision maker) were selected due to prior studies documenting their association with cervical cancer awareness:

7.	Ba DM, Ssentongo P, Musa J, Agbese E, Diakite B, Traore CB, et al. Prevalence and determinants of cervical cancer screening in five sub-Saharan African countries: A population-based study. Cancer Epidemiol. 2021;72:101930.

16.	Lim JN, Ojo AA. Barriers to utilisation of cervical cancer screening in Sub Sahara Africa: a systematic review. Eur J Cancer Care (Engl). 2017;26(1).

18.	Gitonga E, Iseme R, Mutisya R, Kodhiambo M. Cervical cancer knowledge, awareness and related health behaviours amongst women of reproductive age in Kiambu County, Kenya: a cross-sectional study. Health Psychol Behav Med. 2022;10(1):1056-70.

19.	Akokuwebe ME, Idemudia ES, Lekulo AM, Motlogeloa OW. Determinants and levels of cervical Cancer screening uptake among women of reproductive age in South Africa: evidence from South Africa Demographic and health survey data, 2016. BMC Public Health. 2021;21(1):2013.

40.	Okyere J, Aboagye RG, Seidu AA, Asare BY, Mwamba B, Ahinkorah BO. Towards a cervical cancer-free future: women's healthcare decision making and cervical cancer screening uptake in sub-Saharan Africa. BMJ Open. 2022;12(7):e058026.

Next, given a recent DHS study from Jordan documenting an association between intimate partner violence and cervical cancer awareness (21), we included IPV-related variables in this cohort.

21.	Urquhart G, Maclennan SJ, Guntupalli AM. Is there an association between intimate partner violence and the prevalence of cervical cancer screening in Jordan? PLoS One. 2023;18(8):e0290678.

Finally, we included female genital mutilation/circumcision (FGM/C), a risk factor for cervical cancer and a form of gender-based violence, given this represents a potential covariate for cervical cancer awareness, though this has not yet been analyzed in literature to date. Prior studies attribute global disparities in cervical cancer incidence and mortality to gender inequities per the following reference:

7	Ginsburg O, Bray F, Coleman MP, et al. The global burden of women's cancers: a grand challenge in global health. Lancet. 2017 Feb 25;389(10071):847-860. doi: 10.1016/S0140-6736(16)31392-7.

8	Singh GK, Azuine RE, Siahpush M. Global Inequalities in Cervical Cancer Incidence and Mortality are Linked to Deprivation, Low Socioeconomic Status, and Human Development. Int J MCH AIDS. 2012;1(1):17-30. doi: 10.21106/ijma.12. PMID: 27621956; PMCID: PMC4948158.

Please also see the response to Reviewer #1, Question #6.

Additionally, we have modified the title of Tables 2A-C for clarity:

*“Bivariate analysis for association between SES and demographic characteristics and cervical cancer awareness per Mauritania Demographic Health Survey, 2019-2021”*

*“Bivariate analysis for association between SES and demographic characteristics and cervical cancer awareness per Madagascar Demographic Health Survey, 2021”*

*“Bivariate analysis for association between SES and demographic characteristics and cervical cancer awareness per Benin Demographic Health Survey, 2017-2018”*

Conclusion

6. Overall, the paper is well-constructed but requires improvements in data presentation and analysis to provide clearer insights into the correlates of cervical cancer awareness in these countries.

Thank you for this comment. No response indicated. Please also see Responses to Reviewer #3, Question #1, 2, and 4 and Responses to Reviewer #1, Question #5 and 6.
