# Supplementary material for: Correlates of cervical cancer awareness among women aged 30–49 in five sub-Saharan African nations: Evidence from the Demographic and Health Survey (DHS)—2017–2023
Source: PLOS Glob Public Health. 2025 May 7;5(5):e0003344. doi: 10.1371/journal.pgph.0003344 (PMC12057955; doi:10.1371/journal.pgph.0003344)
Supplement: Attachment — Submitted filename: Response_to_reviewers_Jan_3_24_Final.docx [file pgph.0003344.s005.docx]

Response to Reviewer’s Comments

Revision #2

PLOS Global and Public Health

1. **I advise that you address reviewers 1& 2 concerns with regards to what new information is this adding to the current literature, why the five countries and the issue around the age specification for HPV screening**

Thank you for this guidance. We have addressed the comments below.

2. **Reviewer #1: Thank you for the opportunity to reassess the manuscript. I do not know if the wrong file was uploaded. The revised file I assessed has no track changes to indicate changes made. Besides that, I realised that the data was still treated independently rather than appending it.**

Thank you for this comment and for taking the time to review our manuscript. Please see attached for our updated revised “track changes” file, in which all changes are highlighted. Please do not hesitate to reach out directly to the corresponding author at [doliv@uw.edu](mailto:doliv@uw.edu) if you are unable to review or download documentation. We sincerely apologize for any difficulty obtaining or downloading our “tracked changes” document.

We add this sentence to our methodology for clarification:

*“These five countries represent the only SSA nations with DHS questions on cervical cancer awareness published between 2017-2023”* (Lines 148-150)

Regarding appending data, we addended our analyses to include a pooled multi-country analysis on the influence of our covariates on cervical cancer awareness in **Table 3**.

We have added the following sentences in our results:

*“Finally, in our pooled multi-country data set, literacy (AOR=1.25 [1.03-1.51]), greater frequency of television use (AOR=1.47 [1.17-1.84]), mobile telephone ownership (AOR=1.98 [1.36-2.9]), visiting a healthcare facility in the past 12 months (AOR=1.64 [1.25-2.13]) and hormonal contraceptive use (AOR=1.28 [1.08-1.51]) were associated with increased awareness after multivariable adjustment, as seen in Table 3. Contrastingly, women who did not independently make their own healthcare decisions were associated with decreased awareness (AOR=0.86 [0.77-0.97]).”* (Lines 341-347).

3**. My basis for rejecting the manuscript at this point stems from the following:**

**1. There is no novelty in the paper. It adds very little to what we already know about cervical cancer. All of your findings have already been documented in many studies conducted in SSA. (see: https://www.sciencedirect.com/science/article/pii/S1877782121000473; https://www.ajol.info/index.php/pamj/article/view/212679; https://bmjopen.bmj.com/content/12/7/e058026.abstract). So, what new thing is this study adding? I would advise the authors to take time to review literature and identify where there are inconsistencies and capitalize on that. You can even decide to investigate some mediation or moderation effects. But presenting just the correlates adds nothing.**

Thank you for this important comment. We have reviewed the following references to outline prior studies on cervical cancer correlates in SSA. Respectfully, this study fundamentally differs from each of the previously cited studies and other current literature which focuses on factors and covariates of cervical cancer screening, rather than correlates of cervical cancer awareness. This study aims to inform strategies to increasing cervical cancer awareness, a necessary step to increase cervical cancer screening rates, as outlined in paragraph #7 of the discussion section. We also added mediation analyses comparing the relationship between socioeconomic variables on cervical cancer screening using cervical cancer awareness as a mediator.

We added the following mediation analyses to our Results section:

*“Next, to determine the influence of cervical cancer awareness on the relationship between socioeconomic status and cervical cancer screening, additional mediation analyses were performed. The mediation analyses revealed a significant indirect effect of education on cervical cancer screening through cervical cancer awareness in Benin (ACME=0.0059 [0.25-0.01]; p<0.001). The direct effect of education on cervical cancer screening was not significant (ADE=0.1 [-0.003-0.02]; p=0.11). The total effect of education on cervical cancer screening was 0.02, 38% which was mediated through cervical cancer awareness. Similar results were found for the wealth index (ACME=0.002 [0.001-0]; p<0.001, ADE=-0.0003 [-0.01-0.01]; p=0.95) in Benin.*

*Separate mediation analyses demonstrated a significant indirect effect of education on cervical cancer screening through awareness in Cameroon (ACME=0.01 [0.006-0.02], p<0.001; ADE=0.02 [0.007-0.04], p=0.002; Total Effect=0.035, Prop Mediated=0.4), and Madagascar (ACME=0.008 [0.004-0.01], p<0.001; ADE=0.02 [0.01-0.03], p<0.001; Total Effect=0.03, Prop Mediated=0.25). Mediation analyses in Mauritania and Mozambique were not performed since education was not significant in multivariate analysis (Table 2).”* (Lines 360-375)

We also revised the Discussion section as well:

*“Further, our mediations analyses demonstrate that awareness mediates the effects of various socioeconomic variables on cervical cancer screening.”* (Lines 443-444)

Respectfully, we believe that our study adds to existing literature for several reasons:

(1) To the best of our knowledge and based on a PubMed search, this is the first study to describe correlates of cervical cancer awareness – rather than correlates of cervical cancer screening– in Cameroon, Madagascar, Mauritania, and Mozambique using the most recently published DHS analyses.
(2) We report an association between cervical cancer awareness and emotional IPV, the first study to report this relationship between cervical cancer awareness and IPV. This article expands on findings from Urquhart et al in PLosS One (2023), which demonstrated an association between emotional IPV and cervical cancer screening (rather than awareness) from the Jordan DHS.

To clarify this, we added the following sentence:

*“Fourth, women experiencing emotional abuse were paradoxically associated with increased cervical cancer awareness in Cameroon after multivariate adjustment. This finding aligns with one recent study on correlates of cervical cancer screening, though such relationship has not been identified regarding correlates of cervical cancer awareness. (25)”* (Lines 426-428)

(3) We outline the relationship between healthcare decision-making autonomy as a correlate of cervical cancer awareness, which has not been documented currently in literature. This finding has only been documented regarding cervical cancer screening correlates, as cited below in reference #44.

To clarify this, we added the following sentences:

*“Next, lack of independent healthcare decision-making autonomy was associated with decreased cervical cancer awareness across all five countries in our pooled multivariate analysis. This aligns with previously documented associations between healthcare decision-making and cervical cancer screening uptake among six DHS surveys from 2011-2018 in SSA, though such relationship has not been documented as a correlate of cervical cancer awareness. (44)”* (Lines 399-403)

(4) After multivariate adjustment in our pooled data set, various markers of socioeconomic status were not associated with cervical cancer awareness, differing from prior studies on correlates of cervical cancer screening.

To clarify this, we added the following sentences in our discussion:

*“First, literacy and more frequent visits to healthcare facilities were associated with increased cervical cancer awareness after multivariable adjustment. In contrast, several other markers of socioeconomic status – including wealth index, education, and health insurance – were not associated with cervical cancer awareness after multivariate adjustment in our pooled cohort, contrasting with prior studies. (11, 20, 22, 23) Literacy and healthcare utilization may facilitate increased interaction with cervical cancer awareness campaigns. Interventions to reach patients without high literacy or healthcare utilization include community-based lay health advisors (LHAs) and group sessions. (41-43) Notably, socioeconomic variables such as wealth index, education, and literacy, were significant in country-specific multivariable analysis in Benin, Cameroon, and Madagascar, thus caution should be used in interpretation of pooled analyses due to heterogeneity of included countries.”* (Lines 387-397).

(5) We highlight the significance of telecommunication devices (mobile phone ownership and television usage) as statistically significant covariates of cervical cancer awareness. Such data can inform targeted cervical cancer awareness campaigns, which may increase cervical cancer screening uptake.

*“Third, given the positive association between mobile telephone ownership and cervical cancer awareness, cervical cancer awareness campaigns should include communication via mobile telephone (mHealth) given prior studies show effectiveness in low-resource settings. (50) Given that less than 60% of surveyed women owned a cell phone, these results suggest that increasing cell phone ownership in SSA can help advance cervical cancer awareness campaigns.”* (Lines 413-417)

(6) We performed additional mediation analyses per below to examine the mediation of cervical cancer awareness on the relationship between education and cervical cancer screening:

*“Next, to determine the influence of cervical cancer awareness on the relationship between socioeconomic status and cervical cancer screening, additional mediation analyses were performed. The mediation analyses revealed a significant indirect effect of education on cervical cancer screening through cervical cancer awareness in Benin (ACME=0.0059 [0.25-0.01]; p<0.001). The direct effect of education on cervical cancer screening was not significant (ADE=0.1 [-0.003-0.02]; p=0.11). The total effect of education on cervical cancer screening was 0.02, 38% which was mediated through cervical cancer awareness. Similar results were found for the wealth index (ACME=0.002 [0.001-0]; p<0.001, ADE=-0.0003 [-0.01-0.01]; p=0.95) in Benin.*

*Separate mediation analyses demonstrated a significant indirect effect of education on cervical cancer screening through awareness in Cameroon (ACME=0.01 [0.006-0.02], p<0.001; ADE=0.02 [0.007-0.04], p=0.002; Total Effect=0.035, Prop Mediated=0.4), and Madagascar (ACME=0.008 [0.004-0.01], p<0.001; ADE=0.02 [0.01-0.03], p<0.001; Total Effect=0.03, Prop Mediated=0.25). Mediation analyses in Mauritania and Mozambique were not performed since education was not significant in multivariate analysis (Table 2).”* (Lines 360-375)

4. **Why the three selected countries? There are many other countries with DHS (more current) data on cervical cancer screening. So, it beats my imagination why the authors limit themselves to these three if the aim is to investigate the case in SSA.**

Thank you for this important comment. The latest version of the manuscript outlines the latest and most updated DHS from five different SSA nations (Benin, Cameroon, Madagascar, Mauritania, and Mozambique), where correlates of cervical cancer awareness have not been reported, except for Benin.

We agree, ideally additional countries would be included in this analysis. However, these countries represent the only sub-Saharan African nations with DHS surveys with questions on cervical cancer awareness between 2017-2023.

We individually reviewed each DHS survey from 2017-2023 to determine which surveys obtained cervical cancer screening variables. We also reached out to the DHS management team for further clarification in March 2024 to better understand why such cervical cancer screening variables were lacking. They shared DHS questions reflect a lengthy feedback process with in-country Ministries of Health and various Governmental Agencies. As such, questions in DHS surveys vary by year and country.

Please also see Lines 162-172 in our Materials and Methods Section:

*“First, all DHS surveys between 2017-2023 were queried for questions on cervical cancer awareness (e.g., search terms “cervical” OR “cervical cancer” OR “cancer”). Ten countries met initial search criteria for further analysis. Each country’s DHS survey was queried via the DHS publicly accessible Survey Question Database (https://questiondatabase.dhsprogram.com/); additionally, each country’s full survey questionnaire was individually reviewed for questions on cervical cancer awareness via publicly available DHS Summary Reports. Six countries were excluded due to lack of specific questions on cervical cancer awareness including: Burkina Faso (2021); Cote d’Ivorie (2021); Gabon (2019-2021); Ghana (2022); Kenya (2022); Rwanda (2019-2020). Five countries met full inclusion criteria: Benin (2017-2018), Cameroon (2018), Madagascar (2021), Mauritania (2019-2021), and Mozambique (2022-2023).”* (Lines 162-172)

We have also revised our manuscript for clarification:

*“These five countries represent the only SSA nations with DHS questions on cervical cancer awareness published between 2017-2023”* (Lines 148-150).

Importantly, while there are more recently published DHS surveys, unfortunately, these newly published DHS surveys do not include questions on cervical cancer awareness and thus are unable to be included in our data set.

5. **Reviewer #4: Before I thoroughly review the manuscript on the correlates of cervical cancer awareness among five sub-Saharan African nations using data from the Demographic and Health Survey (DHS) from 2017 to 2023, I identified a significant issue related to the age distribution. The authors do not appear to acknowledge the established guidelines for cervical cancer screening, which recommend that screening should begin at age 25 for women living with HIV and at age 30 for those who are HIV-negative. The study includes girls as young as 15, which makes the findings not scientifically sound. Additionally, the upper age limit of 49 years does not align with the recommendations, although this is understandable given that the DHS collects data for women aged 15 to 49. It is crucial for the authors to address this issue, as it has significant implications for overall screening uptake and the regional findings. I believe this oversight could have been mitigated if the authors had reviewed the WHO 2021 guidelines on screening in the background section. I look forward to your explanation regarding this matter. I recommend the analysis be carried out among women aged 30-49 years.**

Thank you for this important and insightful comment. We initially selected a broad age range to better demonstrate discrepancies between various age ranges of women cervical cancer awareness. After further evaluation, however, we opted to revise our data for women aged 30-49 to better reflect that targeted cohort of women who are eligible for cervical cancer screening per 2021 WHO Screening Guidelines. Please see **Table 2** and **Table 3**, along with response to Reviewer #1 above. We have also included the multivariable analyses of the broader age group as **Supplementary Table 1.**

We added the following sentences as limitations to the manuscript as well:

*“Importantly, the 2021 WHO Guidelines note that cervical cancer screening for people living with HIV (PLWH) should be initiated starting at age 25, rather than age 30 for people living without HIV (PLWoH) due to a higher risk of cervical cancer”* (Lines 120-122).

*“Additionally, this study was unable to evaluate the rates of cervical cancer awareness among people living with HIV (PLWH), which is notable given the 2021 WHO Cervical Cancer Screening Guidelines, which recommend initiation of cervical cancer screening at age 25 for PLWH”* (Lines 489-492).
