# Supplementary material for: Correlates of cervical cancer awareness among women aged 30–49 in five sub-Saharan African nations: Evidence from the Demographic and Health Survey (DHS)—2017–2023
Source: PLOS Glob Public Health. 2025 May 7;5(5):e0003344. doi: 10.1371/journal.pgph.0003344 (PMC12057955; doi:10.1371/journal.pgph.0003344)
Supplement: Attachment — Submitted filename: 2025-02-18 Response to Reviewers Document.docx [file pgph.0003344.s006.docx]

Response to Reviewers’ Comments:

EDITOR: Please address reviewer 1 comments as it relates to need to do multi-level regression analysis given that DHS data is hierarchical in nature. Please provide a tangible reason if you think multi-level regression analysis is not necessary in this case.

Reviewer #1:

1. **Thank you for the opportunity to review the revised manuscript. The authors have addressed the initial comments satisfactorily. While there are studies on the topic from individual SSA countries, none have looked at the regional picture. So, this is a good addition to knowledge.**

Thank you for this comment. No response indicated.

2. **Revise the title to read as: "Correlates of cervical cancer awareness among women aged 30-39 years in five sub-Saharan African nations: Evidence from the Demographic and Health Survey"**

Thank you for this comment. We have revised the title of the article accordingly.

New title: “Correlates of cervical cancer awareness among women aged 30-49 in five sub-Saharan African nations: Evidence from the Demographic and Health Survey (DHS)—2017-2023”

3. **I believe that media exposure should be a composite variable. However, if the authors want to keep what they have, then they must provide justifications in the methods section.**

Thank you for this comment. We considered combining “frequency of watching television” with “frequency of listening to the radio” into one composite variable. However, we decided against this for several reasons. First, there are significant inter-country disparities, as shown in Table 1. Next, since television and radio use reflect different consumption patterns and may target different demographics, we wanted to analyze each separately. Additionally, composite variables would lead to a loss of granularity of our data set. Last, we did not want to combine both variables and assume television and radio use contributed equally to a composite variable of media exposure.

Therefore, we opted to not combine forms of media exposure into one composite variable.

We have revised our methodology below:

*“Composite variables were considered (i.e., combining television and radio use into a composite media consumption variables) but this was ultimately not implemented since it would lead to a loss of granularity on the impact of selected variables on cervical cancer awareness”*

4. **The DHS is a hierarchical data. As such, it is important to account for the variations due to clustering. This means a multi-level regression analysis should have been done. Please revise the analysis or provide justification for the approach used, especially when you are working with multiple countries.**

Thank you for this comment. In our initial methodology, we accounted for clustering in the DHS data using adjustment variables to our survey design provided by DHS. We modeled this methodology based on our review of prior studies and our review of the most recently published DHS manual. We created a survey design object, which was inclusive of the cluster variable (v022), stratification variable (v021), and standard sample weights (v005). This adjusted for clustering and aimed to provide representative population estimates.

However, after careful consideration and re-review of the DHS manual and from recommendations from DHS staff in the DHS Program User Forum, we opted to **remove** our multivariate pooled weighted analysis from our manuscript here for several important reasons:

1) Our pooling of five nations across sub-Saharan Africa reflects different time periods (i.e., surveys performed from 2017-2023), varying demographic and social health contexts, and provides an unclear reference population. The differences across time periods and countries makes it challenging to interpret any pooled multi-variable analysis – even when accounting for variation due to clustering.
2) Some covariates, such as wealth index, are unable to be compared in a pooled multi-country analysis (for example, since wealth index is a relative index within each country at the time of the survey, it cannot be pooled into one larger analysis). This is a direct recommendation from DHS Staff Members in the Forum and in published data.
3) While our initial methodology in our multivariate pooled weighted analysis accounted for variations due to clustering, we likely under-estimated total variation and may have provided inaccurate results in our pooled analyses.
4) Senior DHS Officials, in general, strongly recommend **against** conducting pooled multi-country analyses for data that can be appropriately compared at the country-level. In contrast, they recommend performing separate analyses and comparing data. This is based on prior recommendations from senior DHS officers as references cited below in the DHS Forum.

In sum, we opted to remove our pooled multivariable analyses from the manuscript and felt that reporting multivariate weighted analyses for each individual country – as seen in **Table 2** – is more useful, easier to interpret for readers, and more statistically sound. We acknowledge that there are alternative methodologies, such as multi-level regression analyses, that can be performed in similar situations, but we opted against this for the above reasons.

This decision was based on the following references:

Rutstein S. O., Staveteig S., “Making the demographic and health surveys wealth index comparable” (DHS Methodol. Rep., 9, ICF International, Rockville, MD, 2014).

“Calculating fixed and random effects”. The DHS Program User Forum: Discussions regarding the DHS Program data and results. USAID. 10 May 2018. <https://userforum.dhsprogram.com/index.php?t=msg&goto=14875&S=Google>

“Pooled weights”. The DHS Program User Forum: Discussions regarding the DHS Program data and results. USAID. 20 April 2017. <https://userforum.dhsprogram.com/index.php?t=msg&goto=12281&S=Google>

“Pooling cross country IPUMS-DHS data for all available surveys; using svset”. The DHS Program User Forum: Discussions regarding the DHS Program data and results. USAID. 3 July 2019. <https://userforum.dhsprogram.com/index.php?t=msg&goto=17887&S=Google>

To explain this, we added the following sentence to the limitations section:

*“Of note, we opted to not include additional pooled multivariable analyses in this project given the significant heterogeneity across our five selected countries, variation in year of survey, inability to pool certain variables (i.e., wealth index) across countries, unclear reference group, and recommendations from prior DHS staff as described in several online forums and publications.”*

Thank you for your review and our apologies for the lengthy response to this question.

5. **The authors stated: "All models were tested for collinearity with variance inflation factor (VIF) >5 as a barometer for significant collinearity". What was the mean VIF and how did you deal with individual items that had a VIF >5?**

Thank you for this comment.

Our apologies for any confusion – we did not report (nor calculate) the mean VIF in our analyses. Rather, VIFs for each predictor were independently measured to identify significant collinearity. For individual covariates with significant collinearity, as defined by VIF>5, we removed these items from the final analysis.

Finally, in general, multicollinearity is less likely to cause significant statistical problems in this analysis given the large sample size, the stability of regression coefficients, and the known correlation between numerous covariates and cervical cancer awareness.

We revised our methodology below:

*“Each covariate was tested for collinearity, and we utilized a variance inflation factor (VIF) >5 as a barometer for significant collinearity. (35) Covariates with VIF>5 were subsequently removed from our final multivariable model.”*

6. **The authors must document how missing values were handled.**

Thank you for this comment. Missing values were excluded from our analysis via listwise deletion (complete case analysis) based on expert opinion and review of prior of published methodologies on the DHS surveys. All data was weighted as described in our methodology, which ensures the analysis is representative of the population regardless of the missing data.

We revised our methodology below:

*“Missing values were treated via listwise deletion (complete case analysis) and data was weighted above to proportionally reflect the sampled population as noted above.”*
